# The influence of whole-body cryotherapy or winter swimming on the lipid profile and selected adipokines

**DOI:** 10.1186/s13102-023-00744-x

**Published:** 2023-10-19

**Authors:** Bartłomiej Ptaszek, Szymon Podsiadło, Artur Wójcik, Olga Czerwińska-Ledwig, Aneta Teległów

**Affiliations:** 1 Institute of Applied Sciences, University of Physical Education in Krakow, Krakow, 31-571 Poland; 2 Institute of Clinical Rehabilitation, University of Physical Education in Krakow, Krakow, 31-571 Poland; 3 Malopolska Cryotherapy Rehabilitation Center in Krakow, Krakow, 30-036 Poland; 4 Institute of Basic Sciences, University of Physical Education in Krakow, Krakow, 31-571 Poland

**Keywords:** Whole-body cryotherapy, Winter swimming, Lipid profile, Adipokines

## Abstract

**Purpose:**

The aim of this study was to investigate the effect of a series of 20 whole-body cryotherapy (WBC) sessions and 20 winter swimming sessions on the lipid profile and selected adipokines.

**Materials/methods:**

The experimental group consisted of 30 people who underwent a series of WBC treatments and 30 people who underwent a winter swimming. The control group consisted of 30 people - without intervention. Study 1: on the day of the commencement of whole-body cryotherapy / at the beginning of the winter swimming season; and Study 2: after a series of 20 cryotherapy sessions / at the end of the winter swimming season. The control group was also tested twice (4-week break). There were 20 WBC treatments − 5 times a week (4 weeks) and 20 cold water baths - once a week (20 weeks).

**Results:**

A statistically significant increase in the concentration of adiponectin, resistin and leptin in women and resistin and leptin in men was observed after the winter swimming season. Differences were also found in the second study between the groups of women using WBC and the control group, as well as the groups of women swimming and the control group. In men, however, these differences were observed in WBC and the control group. The significance level of α = 0.05 was adopted in the analyzes.

**Conclusions:**

The use of WBC may changes in the lipid profile and selected adipokines in men. Regular winter swimming may changes of selected adipokines in both women and men.

## Introduction

The stimulating effect of low temperatures on the human body brings many benefits appreciated by patients with various diseases who want to reduce their ailments [1, 2], healthy people [3, 4] and athletes who care about good shape (to treat injuries and prevent overtraining) and faster regeneration after exercise (the removal of metabolites and inflammation mediators from damaged tissues) [5]. Cold (local cryotherapy, whole-body cryotherapy, cold water immersion) has an analgesic [6], anti-inflammatory effect [7], reduces swelling [8], causes hyperemia [9] and also has antioxidant properties [10, 11]. Nowadays, both among women and men of all ages, it has become more and more popular to use the health-promoting properties of cold in the form of winter swimming [12] and whole-body cryotherapy (WBC) using cryogenic temperatures (below − 110° C) [13, 14]. Both forms lead to an increase in tolerance to cold, preventing many diseases [15], and also have a positive effect on the mental health, improving general well-being [16, 17] and sometimes even acting as an antidepressant [18]. Regular cold water swimming (CWS) or winter swimming increases immunity [19], strengthens the functions of the cardiovascular system due to “vascular gymnastics” [20], and also has a positive effect on the musculoskeletal system [21]. WBC causes changes in the nervous [22], blood [23], immune [24] and endocrine systems [25]. Muscle contractions induced by exposure to cold, similar to those occurring during exercise, may be an alternative to exercise in preventing or reversing the effects of metabolic diseases such as obesity and type II diabetes [26]. Cholesterol in the human body has many important functions. The most important lipoproteins involved in cholesterol transport are HDL and LDL. Low-density lipoprotein (LDL) transports cholesterol from the liver to other organs, is pro-atherosclerotic and its increased level is observed in connection with obesity, type II diabetes, as well as infectious and inflammatory conditions. High-density lipoprotein (HDL) plays an important role in the reverse transport of cholesterol from peripheral tissues to the liver, demonstrating anti-atherosclerotic effects and has antioxidant, anti-inflammatory and anti-thrombotic properties [27]. It has been shown that both WBC and winter swimming can cause changes in the metabolism of adipose tissue and the lipid profile [20, 28, 29]. Adipose tissue, once perceived only as an energy store, also functions as an endocrine organ secreting bioactive substances - adipokines [30]. There are many indications that exposure of the whole-body to extremely low temperatures causes changes in the metabolism of adipose tissue [28] and the lipid profile [31, 32], while the knowledge about the influence of cold on the levels of selected adipokines remains limited [28]. Previous studies have shown that the reduction of adipose tissue mass increases the level of adipokines in the serum [33] and improves insulin sensitivity, as well as reduces inflammation [34]. Modulation of serum adipokines may lead to weight loss and prevention of comorbidities in obese individuals. However, the implementation of physical activity is not always easy (especially for people with severe obesity), therefore any therapy that affects weight loss and affects the adipokine-lipid profile is recommended [28]. The aim of this study was to investigate the effect of a series of 20 whole-body cryotherapy sessions and 20 winter swimming sessions on the lipid profile and selected adipokines. It was hypothesized that WBC and winter swimming would have a positive effect on the lipid profile (increase in HDL, decrease in LDL, Total Cholesterol (TC) and Triglycerides (TG)) and the concentration of selected adipokines (increase in the level of adiponectin and decrease in the level of asprosin, resistin and leptin). The undoubted advantage of this study is the evaluation of two types of cold factor and the possibility of comparing these results.

## Materials and methods

### Participant characteristics

In total, 104 people applied for the study, and 90 were eventually qualified to participate in the research program (November 2010 – March 2020) (10 people did not meet the inclusion criteria or met the exclusion criteria and 4 people withdrew their consent to the study). The subjects were recruited from the participants of the Kraków Winter Swimming Club “Kaloryfer” (Radiators), patients of the Malopolska Cryotherapy Rehabilitation Center in Krakow and through an advertisement on the Internet. Before the study, each volunteer read the information for the patient about the project and the terms of participation. The participants were qualified for the project by a rehabilitation doctor and a physiotherapist. The respondents declared their eating style as good or very good and the number of meals 4–5 per day (meals were not monitored).

Inclusion criteria: general health at a very good level (without comorbidities); 30–55 years; patient’s consent to participate in the project; no contraindications to cold therapy (i.e., pregnancy, severe hypertension (BP > 180/100), acute or recent myocardial infarction, unstable angina pectoris, arrhythmia, symptomatic cardiovascular disease, cardiac pacemakers, peripheral arterial occlusive disease, venous thrombosis, acute or recent cerebrovascular accident, uncontrolled seizures, Raynaud’s Syndrome, fever, tumor disease, symptomatic lung disorders, bleeding disorders, severe anemia, infection, cold allergy, and acute kidney and urinary tract diseases). Exclusion criteria: change of eating style before or during the project; participating in other forms of physiotherapy or training before or during the project. All respondents did not practice any sport professionally.

The experimental group consisted of 15 women and 15 men who underwent a series of whole-body cryotherapy treatments and 15 women and 15 men who underwent a winter swimming. The control group consisted of 15 women and 15 men - without intervention (controlled assignment to groups). Body weight and body composition were assessed using the Tanita BC 418 MA and examined before the treatments began (Tables 1, 2, 3 and 4).

**Table 1:** General characteristics of the respondents and comparison within groups WOMEN-CRYO, WOMEN-WS, WOMEN-CONTROL.

Characteristics	WOMEN-CRYO n = 15	WOMEN-WS n = 15	(p)	WOMEN-CRYO n = 15	WOMEN-CONTROL n = 15	(p)	WOMEN-WS n = 15	WOMEN-CONTROL n = 15	(p)
Age [years]	37.96 ± 6.34	44,71 ± 10.19	0.003*	37.96 ± 6.34	35,86 ± 7.61	0.374	44,71 ± 10.19	35,86 ± 7.61	0.000*
Body height [cm]	169.16 ± 5.96	164,18 ± 5.63	0.005*	169.16 ± 5.96	167,56 ± 7.64	0.388	164,18 ± 5.63	167,56 ± 7.64	0.055
Body mass [kg]	72.69 ± 13.40	66,35 ± 8.51	0.047*	72.69 ± 13.40	66,19 ± 13.79	0.054	66,35 ± 8.51	66,19 ± 13.79	0.961
Body mass index [kg/m^2^]	25.43 ± 4.78	24,66 ± 3.33	0.430	25.43 ± 4.78	23,54 ± 4.49	0.068	24,66 ± 3.33	23,54 ± 4.49	0.251
Fat [%]	31.13 ± 6.73	29,75 ± 5.43	0.076	31.13 ± 6.73	25,18 ± 6.86	0.002*	29,75 ± 5.43	25,18 ± 6.86	0.134
Lean body mass [kg]	23.39 ± 5.57	20,07 ± 4.05	0.095	23.39 ± 5.57	17,27 ± 7.46	0.842	20,07 ± 4.05	17,27 ± 7.46	0.144
Total body water [kg]	49.31 ± 4.08	46,29 ± 2.96	0.094	49.31 ± 4.08	48,93 ± 5.46	0.839	46,29 ± 2.96	48,93 ± 5.46	0.144

*: statistically significant (p < 0.05)

**Table 2:** General characteristics of the respondents and comparison within groups MEN-CRYO, MEN-WS, MEN-CONTROL.

Characteristics	MEN-CRYO n = 15	MEN -WS n = 15	(p)	MEN -CRYO n = 15	MEN -CONTROL n = 15	(p)	MEN -WS n = 15	MEN -CONTROL n = 15	(p)
Age [years]	35,93 ± 7.90	46,64 ± 13.72	0.000*	35,93 ± 7.90	29,60 ± 5.14	0.008*	46,64 ± 13.72	29,60 ± 5.14	0.000*
Body height [cm]	179,73 ± 9.36	178,67 ± 7.12	0.556	179,73 ± 9.36	182,73 ± 7.04	0.106	178,67 ± 7.12	182,73 ± 7.04	0.025*
Body mass [kg]	80,70 ± 11.99	86,65 ± 17.64	0.069	80,70 ± 11.99	83,33 ± 11.01	0.434	86,65 ± 17.64	83,33 ± 11.01	0.309
Body mass index [kg/m^2^]	25,03 ± 3.60	26,95 ± 3.91	0.054	25,03 ± 3.60	25,02 ± 3.68	0.992	26,95 ± 3.91	25,02 ± 3.68	0.053
Fat [%]	18,68 ± 3.67	20,83 ± 6.06	0.172	18,68 ± 3.67	14,62 ± 5.89	0.013*	20,83 ± 6.06	14,62 ± 5.89	0.000*
Lean body mass [kg]	65,28 ± 8.36	67,90 ± 10.43	0.157	65,28 ± 8.36	70,71 ± 6.81	0.005*	67,90 ± 10.43	70,71 ± 6.81	0.130
Total body water [kg]	47,79 ± 6.12	49,71 ± 7.64	0.156	47,79 ± 6.12	51,77 ± 4.98	0.005*	49,71 ± 7.64	51,77 ± 4.98	0.129

*: statistically significant (p < 0.05)

**Table 3:** Shapiro-Wilk test for normality of characteristics data in women group.

Characteristics	WOMEN-CRYO n = 15		WOMEN-WS n = 15		WOMEN-CONTROL n = 15	
	W	(p)	W	(p)	W	(p)
Age [years]	0,944	0,118	0,881	0,000*	0,942	0,106
Body height [cm]	0,895	0,006*	0,921	0,010*	0,925	0,038*
Body mass [kg]	0,944	0,118	0,930	0,020*	0,874	0,002*
Body mass index [kg/m^2^]	0,933	0,059	0,942	0,052	0,773	0,000*
Fat [%]	0,976	0,734	0,945	0,064	0,949	0,162
Lean body mass [kg]	0,936	0,072	0,943	0,053	0,951	0,185
Total body water [kg]	0,936	0,071	0,944	0,060	0,951	0,183

*: statistically significant (p < 0.05)

**Table 4:** Shapiro-Wilk test for normality of characteristics data in men group.

Characteristics	MEN-CRYO n = 15		MEN-WS n = 15		MEN-CONTROL n = 15	
	W	(p)	W	(p)	W	(p)
Age [years]	0,929	0,047*	0,879	0,001*	0,914	0,019*
Body height [cm]	0,951	0,180	0,961	0,270	0,933	0,062
Body mass [kg]	0,919	0,026*	0,908	0,007*	0,973	0,625
Body mass index [kg/m^2^]	0,880	0,002*	0,927	0,025*	0,910	0,015*
Fat [%]	0,953	0,203	0,954	0,163	0,956	0,244
Lean body mass [kg]	0,956	0,251	0,950	0,124	0,954	0,227
Total body water [kg]	0,956	0,255	0,949	0,121	0,954	0,229

*: statistically significant (p < 0.05)

### Ethics approval and consent to participate

The study was approved by the Bioethical Committee of the District Medical Chamber in Krakow (194/KBL/OIL/2019, 09 September 2019) and was conducted in accordance with the assumptions of the Declaration of Helsinki. Informed consent was obtained from all subjects involved in the study.

### Analysis of biochemical blood indices

In order to analyze blood parameters, venous blood was collected twice: on the day of commencement of systemic cryotherapy / at the beginning of the winter bathing season (November); and after a series of 20 cryotherapy treatments / at the end of the winter bathing season (March). The control group was also tested twice (4-week break). Blood from the subjects was collected into tubes with coagulation activator - for serum testing (6 ml). Blood was collected by a laboratory diagnostician in accordance with applicable standards.

TC, TG, HDL, LDL were determined using a colorimetric test (BioMaxima, Poland). The tests were performed using the Cobas c311 501 analyzer (Roche Diagnostics). Asprosin, adiponectin, resistin and leptin were determined in the serum. The indices were investigated with photometric tests: Human asprosin ELISA Kit 201-12-7691 - Shanghai Sunred Biological Technology Co., China; Adiponectin ELISA E09 and Resistin ELISA E50 - Mediagnost Gesellschaft für Forschung und Herstellung von Diagnostika GmbH, Germany; Leptin Sandwich ELISA EIA-2395 - DRG Instruments GmbH, Germany. Procedure in accordance with the manufacturer’s recommendations.

### Description of the intervention

Whole-body cryotherapy (WBC) was carried at the Malopolska Cryotherapy Rehabilitation Center in Krakow. Parameters in the cryochamber were as follows: atrium temperature: −60 °C and chamber temperature: −120 °C. The treatments were performed in a Wroclaw-type cryochamber wherein liquid nitrogen was the refrigerant cooling. The time of a single WBC session during study period was respectively: 1.5 min (1st treatment); 2 min (2nd treatments); 3 min (3rd–20th treatments). One treatment a day was performed (every day in the same time period, 3:00 p.m.–5:00 p.m.). There were 20 treatments in total, and they were performed 5 times a week, in the winter months - the most commonly used WBC protocol [35].

A single winter swimming session lasted 4 min (November - March) − 1x / week − 20 baths per season. Cold water swimming parameters: water temperature 3–6 °C. Bath was carried at Kraków Winter Swimming Club “Kaloryfer” (Radiators) - Bagry Lagoon. The bath consisted of whole body immersion in a cold lake (excluding the head). During the bath, the subjects moved their upper and lower limbs while submerged. During the bath, the participants of the study were dressed in swimsuits, a winter hat and special water shoes. Before entering the water, the subjects performed a short (5-minute) warm-up of moderate intensity. The most frequently used winter swimming protocol was used.

Completely different but most commonly used WBC and CWS standard protocols are presented.

### Statistical analysis

Descriptive statistics were determined: mean (x) as well as standard deviation (SD). The normality of distributions was verified with the Shapiro-Wilk test. Data distribution analysis was performed using ANOVA test or Kruskal-Wallisa test. The applied tests verified two-sided hypotheses. The significance level of p = 0.05 was adopted in the analyzes. In order to determine sample size, the formula for the minimum sample size was used, in which the confidence interval was 95%, the fraction size of 0,5 and the maximum error of 5% were assumed. The analyses were performed with the use of the Statistica 13 package (Tibco Software Inc., USA).

## Results

A statistically significant increase in the concentration of adiponectin, resistin and leptin in women and resistin and leptin in men was observed after the winter swimming season (Tables 5, 6, 7, 8, 9 and 10). Differences were also found in the second study between the groups of women using WBC and the control group (adiponectin and resistin) and the groups of women swimming and the control group (adiponectin). In men, however, these differences were observed between the second examination in the WBC and control groups (LDL, adiponectin). Tables 11, 12 and 13 present the average values of the tested parameters along with the standard deviation.

**Table 5:** Intragroup comparisons of mean values of indicators - whole-body cryotherapy.

Parameter	WOMEN-CRYO		MEN-CRYO	
	Study 1	Study 2	Study 1	Study 2
TC (mmol/L)	4.60 ± 0.82	4.63 ± 0.81	4.94 ± 1.04	4.81 ± 1.11
TG (mmol/L)	0.99 ± 0.71	1.11 ± 0.60	1.33 ± 0.89	1.19 ± 0.85
HDL (mmol/L)	1.66 ± 0.36	1.61 ± 0.35	1.41 ± 0.30	1.52 ± 0.30
LDL (mmol/L)	2.99 ± 0.92	3.04 ± 0.87	3.42 ± 1.03	3.31 ± 1.08
Asprosin (ng/mL)	80.61 ± 93.95	79.67 ± 98.78	72.71 ± 102.03	89.16 ± 126.48
Adiponectin (µg/mL)	1.43 ± 0.68	1.41 ± 0.73	1.33 ± 0.62	1.08 ± 0.61
Resistin (ng/mL)	1.37 ± 0.32	1.25 ± 0.32	1.15 ± 0.38	1.08 ± 0.28
Leptin (ng/mL)	0.99 ± 0.42	1.11 ± 0.40	0.60 ± 0.27	0.53 ± 0.18

**Table 6:** Intragroup comparisons of mean values of indicators - winter swimming.

Parameter	WOMEN-WS		MEN-WS	
	Study 1	Study 2	Study 1	Study 2
TC (mmol/L)	4.98 ± 0.65	4.64 ± 0.89	5.11 ± 0.78	4.85 ± 0.69
TG (mmol/L)	0.98 ± 0.51	0.84 ± 0.28	1.30 ± 1.07	1.16 ± 0.68
HDL (mmol/L)	1.99 ± 0.42	1.91 ± 0.52	1.65 ± 0.71	1.61 ± 0.68
LDL (mmol/L)	3.13 ± 0.55	2.82 ± 0.69	3.53 ± 0.71	3.25 ± 0.70
Asprosin (ng/mL)	47.83 ± 94.05	34.94 ± 74.82	44.76 ± 89.93	32.16 ± 73.10
Adiponectin (µg/mL)	1.19 ± 1.20	2.90 ± 0.68	0.77 ± 0.29	1.41 ± 0.54
Resistin (ng/mL)	1.25 ± 0.51	1.83 ± 0.38	1.09 ± 0.28	1.55 ± 0.34
Leptin (ng/mL)	0.76 ± 0.24	1.12 ± 0.31	0.48 ± 0.17	0.87 ± 0.36

**Table 7:** Intragroup comparisons of mean values of indicators - without intervention.

Parameter	WOMEN-CONTROL		MEN-CONTROL	
	Study 1	Study 2	Study 1	Study 2
TC (mmol/L)	4.61 ± 0.80	4.45 ± 0.59	4.29 ± 0.80	4.20 ± 0.76
TG (mmol/L)	1.02 ± 0.41	0.98 ± 0.46	0.98 ± 0.41	1.13 ± 0.60
HDL (mmol/L)	1.88 ± 0.34	1.80 ± 0.47	1.50 ± 0.34	1.48 ± 0.34
LDL (mmol/L)	2.68 ± 0.78	2.59 ± 0.60	2.77 ± 0.78	2.67 ± 0.68
Asprosin (ng/mL)	86.30 ± 104.05	85.74 ± 102.56	54.33 ± 104.05	42.39 ± 70.81
Adiponectin (µg/mL)	2.11 ± 0.81	2.33 ± 0.82	1.62 ± 0.81	1.64 ± 0.81
Resistin (ng/mL)	1.70 ± 0.31	1.72 ± 0.47	1.46 ± 0.31	1.57 ± 0.45
Leptin (ng/mL)	1.26 ± 0.30	1.24 ± 0.37	0.72 ± 0.30	0.73 ± 0.25

**Table 8:** Shapiro-Wilk test for normality of parameters data in women groups.

Parameter	WOMEN-CRYO n = 15		WOMEN-WS n = 15		WOMEN-CONTROL n = 15	
	W	(p)	W	(p)	W	(p)
TC (mmol/L)	0,919	0,025*	0,951	0,344	0,983	0,903
TG (mmol/L)	0,833	0,000*	0,900	0,030*	0,887	0,004*
HDL (mmol/L)	0,940	0,093	0,955	0,403	0,948	0,149
LDL (mmol/L)	0,845	0,000*	0,962	0,543	0,932	0,058
Asprosin (ng/mL)	0,796	0,000*	0,539	0,000*	0,826	0,000*
Adiponectin (µg/mL)	0,946	0,134	0,846	0,002*	0,912	0,017*
Resistin (ng/mL)	0,946	0,139	0,949	0,303	0,874	0,002*
Leptin (ng/mL)	0,918	0,024*	0,957	0,448	0,962	0,357

*: statistically significant (p < 0.05)

**Table 9:** Shapiro-Wilk test for normality of parameters data in men groups.

Parameter	MEN-CRYO n = 15		MEN-WS n = 15		MEN-CONTROL n = 15	
	W	(p)	W	(p)	W	(p)
TC (mmol/L)	0,944	0,119	0,956	0,475	0,953	0,210
TG (mmol/L)	0,726	0,000*	0,680	0,000*	0,802	0,000*
HDL (mmol/L)	0,961	0,338	0,862	0,008*	0,956	0,249
LDL (mmol/L)	0,969	0,538	0,957	0,499	0,944	0,118
Asprosin (ng/mL)	0,664	0,000*	0,522	0,000*	0,584	0,000*
Adiponectin (µg/mL)	0,899	0,008*	0,935	0,199	0,957	0,259
Resistin (ng/mL)	0,946	0,136	0,918	0,090	0,968	0,510
Leptin (ng/mL)	0,840	0,000*	0,866	0,010*	0,953	0,204

*: statistically significant (p < 0.05)

**Table 10:** Intragroup and intergroup comparisons of mean values of indicators in women (CRYO and CONTROL).

Parameter	WOMEN-CRYO			WOMEN-CONTROL			CRYO/CONTROL (p –ANOVA/Kruskal-Wallisa)	
	Study 1	Study 2	(p)	Study 1	Study 2	(p)	Study 1	Study 2
TC (mmol/L)	4.60 ± 0.82	4.63 ± 0.81	0.097	4.94 ± 1.04	4.81 ± 1.11	0.583	0.966	0.533
TG (mmol/L)	0.99 ± 0.71	1.11 ± 0.60	0.629	1.33 ± 0.89	1.19 ± 0.85	0.883	0.911	0.605
HDL (mmol/L)	1.66 ± 0.36	1.61 ± 0.35	0.769	1.41 ± 0.30	1.52 ± 0.30	0.603	0.179	0.263
LDL (mmol/L)	2.99 ± 0.92	3.04 ± 0.87	0.849	3.42 ± 1.03	3.31 ± 1.08	0.765	0.294	0.125
Asprosin (ng/mL)	80.61 ± 93.95	79.67 ± 98.78	0.979	72.71 ± 102.03	89.16 ± 126.48	0.987	0.871	0.863
Adiponectin (µg/mL)	1.43 ± 0.68	1.41 ± 0.73	0.950	1.33 ± 0.62	1.08 ± 0.61	0.431	0.001*	0.000*
Resistin (ng/mL)	1.37 ± 0.32	1.25 ± 0.32	0.309	1.15 ± 0.38	1.08 ± 0.28	0.874	0.024*	0.000*
Leptin (ng/mL)	0.99 ± 0.42	1.11 ± 0.40	0.317	0.60 ± 0.27	0.53 ± 0.18	0.896	0.026*	0.266

*: statistically significant (p < 0.05)

**Table 11:** Intragroup and intergroup comparisons of mean values of indicators in women (WS and CONTROL).

Parameter	WOMEN-WS			WOMEN-CONTROL			WS/CONTROL (p –ANOVA/Kruskal-Wallisa)	
	Study 1	Study 2	(p)	Study 1	Study 2	(p)	Study 1	Study 2
TC (mmol/L)	4.98 ± 0.65	4.64 ± 0.89	0.318	4.94 ± 1.04	4.81 ± 1.11	0.583	0.252	0.562
TG (mmol/L)	0.98 ± 0.51	0.84 ± 0.28	0.612	1.33 ± 0.89	1.19 ± 0.85	0.883	0.885	0.579
HDL (mmol/L)	1.99 ± 0.42	1.91 ± 0.52	0.658	1.41 ± 0.30	1.52 ± 0.30	0.603	0.519	0.518
LDL (mmol/L)	3.13 ± 0.55	2.82 ± 0.69	0.359	3.42 ± 1.03	3.31 ± 1.08	0.765	0.152	0.469
Asprosin (ng/mL)	47.83 ± 94.05	34.94 ± 74.82	0.753	72.71 ± 102.03	89.16 ± 126.48	0.987	0.315	0.185
Adiponectin (µg/mL)	1.19 ± 1.20	2.90 ± 0.68	0.000*	1.33 ± 0.62	1.08 ± 0.61	0.431	0.002*	0.051*
Resistin (ng/mL)	1.25 ± 0.51	1.83 ± 0.38	0.000*	1.15 ± 0.38	1.08 ± 0.28	0.874	0.003*	0.450
Leptin (ng/mL)	0.76 ± 0.24	1.12 ± 0.31	0.013*	0.60 ± 0.27	0.53 ± 0.18	0.896	0.000*	0.339

*: statistically significant (p < 0.05)

**Table 12:** Intragroup and intergroup comparisons of mean values of indicators in men (CRYO and CONTROL).

Parameter	MEN-CRYO			MEN-CONTROL			CRYO/CONTROL (p –ANOVA/Kruskal-Wallisa)	
	Study 1	Study 2	(p)	Study 1	Study 2	(p)	Study 1	Study 2
TC (mmol/L)	4.94 ± 1.04	4.81 ± 1.11	0.651	4.29 ± 0.80	4.20 ± 0.76	0.766	0.029*	0.043*
TG (mmol/L)	1.33 ± 0.89	1.19 ± 0.85	0.577	0.98 ± 0.41	1.13 ± 0.60	0.543	0.149	0.780
HDL (mmol/L)	1.41 ± 0.30	1.52 ± 0.30	0.504	1.50 ± 0.34	1.48 ± 0.34	0.947	0.612	0.820
LDL (mmol/L)	3.42 ± 1.03	3.31 ± 1.08	0.715	2.77 ± 0.78	2.67 ± 0.68	0.739	0.026*	0.028*
Asprosin (ng/mL)	72.71 ± 102.03	89.16 ± 126.48	0.639	54.33 ± 104.05	42.39 ± 70.81	0.733	0.600	0.184
Adiponectin (µg/mL)	1.33 ± 0.62	1.08 ± 0.61	0.345	1.62 ± 0.81	1.64 ± 0.81	0.947	0.285	0.038*
Resistin (ng/mL)	1.15 ± 0.38	1.08 ± 0.28	0.646	1.46 ± 0.31	1.57 ± 0.45	0.436	0.023*	0.000
Leptin (ng/mL)	0.60 ± 0.27	0.53 ± 0.18	0.521	0.72 ± 0.30	0.73 ± 0.25	0.901	0.347	0.089

*: statistically significant (p < 0.05)

**Table 13:** Intragroup and intergroup comparisons of mean values of indicators in men (WS and CONTROL).

Parameter	MEN-WS			MEN-CONTROL			WS/CONTROL (p –ANOVA/Kruskal-Wallisa)	
	Study 1	Study 2	(p)	Study 1	Study 2	(p)	Study 1	Study 2
TC (mmol/L)	5.11 ± 0.78	4.85 ± 0.69	0.477	4.29 ± 0.80	4.20 ± 0.76	0.766	0.015*	0.054
TG (mmol/L)	1.30 ± 1.07	1.16 ± 0.68	0.640	0.98 ± 0.41	1.13 ± 0.60	0.543	0.242	0.907
HDL (mmol/L)	1.65 ± 0.71	1.61 ± 0.68	0.831	1.50 ± 0.34	1.48 ± 0.34	0.947	0.398	0.502
LDL (mmol/L)	3.53 ± 0.71	3.25 ± 0.70	0.436	2.77 ± 0.78	2.67 ± 0.68	0.739	0.020*	0.075
Asprosin (ng/mL)	44.76 ± 89.93	32.16 ± 73.10	0.769	54.33 ± 104.05	42.39 ± 70.81	0.733	0.807	0.794
Adiponectin (µg/mL)	0.77 ± 0.29	1.41 ± 0.54	0.053	1.62 ± 0.81	1.64 ± 0.81	0.947	0.005*	0.461
Resistin (ng/mL)	1.09 ± 0.28	1.55 ± 0.34	0.006*	1.46 ± 0.31	1.57 ± 0.45	0.436	0.016*	0.909
Leptin (ng/mL)	0.48 ± 0.17	0.87 ± 0.36	0.008*	0.72 ± 0.30	0.73 ± 0.25	0.901	0.081	0.297

*: statistically significant (p < 0.05)

## Discussion

Two groups of 30 people (15 women and 15 men) took part in our experiment, one of whom underwent a series of 20 WBC treatments, and the other - winter swimming / CWS. The third group (control), also consisting of 15 women and 15 men, remained without intervention. In the group of men who underwent a series of 20 WBC treatments, we observed significantly different levels of LDL in the blood in the second study compared to the second study in the control group of men. Lubkowska et al. (2019), described in their study the beneficial effect of systemic cryotherapy on the lipid profile of young, healthy men (increase in HDL concentration and decrease in LDL after 20 and 30 sessions) [9]. Also in other studies, which checked whether the positive effect of exposure to cryogenic temperature depends on their number in a series, a significant increase in HDL concentration and a decrease in LDL was noticed in physically active, healthy men after 20 WBC treatments [36]. It is suggested that even 20 WBC sessions significantly change the lipid profile in healthy people, which was only slightly reflected in our experiment. These observations may explain the lack of significant changes in HDL concentration in the results of some studies in which the number of sessions used (< 20) may not be sufficient [23, 37, 38]. Some authors observed non-statistically significant HDL upward trends after WBC [29], also in combination with exercise [39, 40] in obese subjects. There are many factors that influence lipid profile, including: body weight, female gender, physical activity, consumption alcohol, estrogens, and glucocorticoids; perhaps that is why the changes were only observed in the group of men [36]. Lipid ratio, like TC/HDL cholesterol and the LDL cholesterol/HDL cholesterol ratio correlate with cardiovascular disease. A significant reduction in the proportion of LDL cholesterol to HDL cholesterol and of TC to HDL cholesterol seems beneficial enough to consider whole-body cryostimulation a useful method of atherosclerosis prevention. HDL, the beneficial form of blood cholesterol, protects the artery wall against atherosclerosis and cardiovascular disease [36, 41].

The levels of HDL, LDL, TC and TG in the participants of our study did not change significantly In any of the studied groups after the end of the winter swimming season and 20 sessions of WBC. Other studies can find similar data. Wang et al. (2019), observing middle-aged and elderly people swimming in winter for a year, observed a significant increase in HDL with no significant difference between LDL, TC and TG [20]. Bryczkowska et al. (2018), while administering 30 WBC sessions to MS patients, did not notice any changes in the lipid profile, including the levels of LDL, TC and TG [42]. No significant changes in the lipid profile were also noticed by Misiorek et al. (2018) in older women after 10 WBC treatments [43]. There are also some studies that showed that WBC procedures could decrease lipid profile [25, 29, 32]. In the study [37] that included healthy men, it was reported that the levels of T-Chol and LDL-Chol decreased significantly after 10 procedures of WBC with exercise, but the levels of HDL did not change significantly. And it was also confirmed by meta-analysis [29], which showed that WBC may exert beneficial effects on the lipid profile in terms of lowering the levels of TC, LDL, and TG. The same effect was also observed in patients with ankylosing spondylitis [32]. The explanation of these results may be the fact that WBC followed by exercise improves lipid profile better than only WBC procedures by additive effect. There are various mechanisms that may explain the beneficial effect of WBC on the lipid profile. A study by Vallerand et al. (1989) showed that the human body exposed to cold reacts with an increase in lipid metabolism, and brown adipose tissue plays a major role in the metabolism of TG [44]. Moreover, Yizhen et al. (2015) found that brown adipose tissue generates heat by breaking down TG stored in intercellular lipid droplets during exposure to low temperature to maintain a stable temperature in mice [45]. It is also worth noting that the improvement of the lipid profile after WBC may be associated with a reduction in inflammation. The inflammatory response of tissues to WBC is identified with a decrease in serum C-reactive protein and seromucoid levels, as well as an increase in anti-inflammatory cytokines [46, 47].

Adipose tissue produces biologically active substances that act both locally and systemically, known as adipokines [30, 48]. One of them - leptin (LEP), known as the “satiety hormone”, regulates the appetite and energy balance of the body [49]. In this experiment, in women and men swimming in winter, an increase in leptin levels was observed after the season and a tendency to increase in women after WBC and decrease in men after WBC. In their studies, Pilch et al. (2022) noticed a significant decrease in the concentration of this adipokine in men between 10 and 20 sessions of WBC, but it concerned obese people [28]. As it is known, elevated LEP levels occurring in obese people are associated with the insensitivity to its endogenous production. These authors also concluded that obesity in humans is more likely to be due to central mechanisms that regulate food intake and energy expenditure than to defective signaling by adipocytes to these central mechanisms. [50]. A decrease in LEP levels has also been reported by other authors who examined people with diagnosed type II diabetes or metabolic syndrome after 10 WBC treatments used once or twice a week [51] and middle-aged women swimming in cold water twice a week for 7 consecutive months [52]. Swimming in cold water should be seen not only as a cold stimulus, but also as physical exercise. An analysis of studies by Fedewa et al. (2018) indicates a decrease in leptin levels after a physical exercise program regardless of age and gender. The frequency of our training in the water was probably too low to achieve a similar effect [53]. Previous studies show that obese people are resistant to LEP [54]. Therefore, any treatment that results in a decrease in the level of LEP or hypersensitivity to LEP may be a good strategy to prevent lifestyle diseases, very often associated with obesity [55]. In our study, a trend towards lower LEP levels was observed in the group of men using WBC. This decrease may be beneficial given the effects of the treatments on muscle tremor and glucose uptake and metabolism [55].

ADP increased in females after the winter swimming season. Adiponectin increases insulin sensitivity, lowers the level of circulating lipids and protects against atherosclerosis [56, 57], and low levels of it are found in people with obesity, insulin resistance and type II diabetes [58]. The adiponectin pathway may play an important role in the development of drugs for type II diabetes and other obesity-related diseases that are affected by insulin resistance. It seems that adiponectin may increase insulin sensitivity mainly by improving lipid and glucose metabolism. Inconsistent support in the literature exists for increasing adiponectin levels after short-term exposure to robust aerobic or resistance training of moderate-to-high intensities [59]. Many studies report on the anti-inflammatory properties of WBC, as evidenced by the reduction of, for example, the level of TNF-alpha, which in turn inhibits the expression of the ADP gene in adipocytes [60].

In addition to changes in ADP and LEP, cold water swimming also led to increases in resistin (REZ) concentrations in women. Ziemann et al. (2013) examining the effect of 10 WBC treatments on obese men found an increase in REZ level in one of the groups with no changes in LEP and ADP [61]. The primary function of resistin in the body is to maintain normal blood glucose levels during starvation. In the case of chronic inflammation, higher levels of resistin are noted [49], and prolonged elevated levels in the blood predispose to the development of type II diabetes [62]. Increased resistin levels after the winter swimming season may be associated with an induced general mild pro-inflammatory response of the body. According to the Cobbold (2019) review, plasma resistin levels decreased following aerobic endurance exercise and/or resistance training plans in obese and/or insulin resistant individuals [63].

In the studies of other authors, it can be noted that some of them did not show changes in the concentration of adipokines: leptin [39, 61], adiponectin [28, 39, 61] and resistin [39] after alone WBC or in combination with exercise. The varied results of the conducted experiments do not give a clear answer to the topic of the influence of cold on selected adipokines and may indicate the complexity of the issue and the need to continue research in different treatment configurations in different groups of patients. Considering that obesity is a global epidemic, the incidence of which is increasing in most societies, and knowing that it can gradually cause or worsen other diseases such as type II diabetes, hypertension, cardiovascular disease or mental disorders, it is reasonable is a constant search for solutions to reduce this phenomenon [64]. In addition to the undoubted benefits of WBC and CWS, such as analgesic and anti-inflammatory effects, confirmed by many reports, it seems necessary to continue research into the effects of these treatments on adipose tissue in order to obtain the necessary data. The developed procedures could be an alternative to physical effort in the fight against the effects of the disease for people with reduced mobility.

To the best of our knowledge, this study is the first to evaluate the effects of WBC and winter swimming on the lipid profile and adipokines levels in men and women. No pathological or harmful changes were observed after the use of WBC and winter swimming.

Study Limitation - The present experiment was not without disadvantages, related to even a small number of participants or the lack of a uniform diet, but it undoubtedly showed the influence of WBC and CWS on changes in the lipid profile and selected adipokines in women and men. When analyzing the general characteristics of the subjects, differences between the groups were observed. While the subjects were similar in age, BMI and lifestyle, the results may reflect differences in body composition. Studies should be continued in groups of patients with different BMI. Two completely different protocols, WBC and CWS, are shown and cannot be used interchangeably.

## Conclusions

The use of whole-body cryotherapy may changes in the lipid profile and selected adpipokines in men. Regular winter swimming may changes of selected adipokines in both women and men.

## Data Availability

All data generated or analyzed during this study are included in this published article.
